# [Retracted] Downregulation of long non‑coding RNA GAS5 promotes cell proliferation, migration and invasion in esophageal squamous cell carcinoma

**DOI:** 10.3892/ol.2024.14372

**Published:** 2024-04-02

**Authors:** Ke Ke, Zhanwen Sun, Zhengjun Wang

Oncol Lett 16: 1801–1808, 2018; DOI: 10.3892/ol.2018.8797

Following the publication of this article, a concerned reader drew to the Editor's attention that, for the Transwell migration and invasion assay experiments shown in Fig. 4B and D on p. 1806, an unexpected area of similarity was identified in terms of the cellular data comparing the two ‘pc-NC’ data panels, where the results from differently performed experiments were intended to have been shown, although the areas immediately surrounding this area featured comparatively different distributions of cells. In addition, the western blots in this article were presented with atypical, unusually shaped and possibly anomalous protein bands in many cases.

After having conducted an internal investigation, the Editor of *Oncology Letters* has reached the conclusion that the potentially anomalous data in this paper were unlikely to have arisen by coincidence. Therefore, on the grounds of a lack of confidence in the integrity of these data, the Editor has decided that the article should be retracted from the publication. The authors were asked for an explanation to account for these concerns, but the Editorial Office did not receive a reply. The Editor apologizes to the readership for any inconvenience caused, and thanks the concerned reader for drawing this matter to our attention.

